# Comparative transcriptomics provide insight into the morphogenesis and evolution of fistular leaves in *Allium*

**DOI:** 10.1186/s12864-016-3474-8

**Published:** 2017-01-10

**Authors:** Siyuan Zhu, Shouwei Tang, Zhijian Tan, Yongting Yu, Qiuzhong Dai, Touming Liu

**Affiliations:** Institute of Bast Fiber Crops and Center of Southern Economic Crops, Chinese Academy of Agricultural Sciences, Changsha, China

**Keywords:** Allium, Transcriptome, Fistular leaf, Selective pattern, Programmed cell death

## Abstract

**Background:**

Fistular leaves frequently appear in *Allium* species, and previous developmental studies have proposed that the process of fistular leaf formation involves programmed cell death. However, molecular evidence for the role of programmed cell death in the formation of fistular leaf cavities has yet to be reported.

**Results:**

In this study, we characterized the leaf transcriptomes of nine *Allium* species, including six fistular- and three solid-leaved species. In addition, we identified orthologous genes and estimated their Ka and Ks values, in order to ascertain their selective pattern. Phylogenetic analysis based on the transcriptomes revealed that *A. tuberosum* was the most ancestral among the nine species, and analysis of orthologous genes between *A. tuberosum* and the other eight species indicated that 149 genes were subject to positive selection; whereas >3000 had undergone purifying selection in each species.

**Conclusions:**

We found that many genes that are potentially related to programmed cell death either exhibited rapid diversification in fistular-leaved species, or were conserved in solid-leaved species in evolutionary history. These genes potentially involved in programmed cell death might play important roles in the formation of fistular leaf cavities in *Allium*, and the differing selection patterns in fistular- and solid-leaved species may be responsible for the evolution of fistular leaves.

**Electronic supplementary material:**

The online version of this article (doi:10.1186/s12864-016-3474-8) contains supplementary material, which is available to authorized users.

## Background

As one of the largest genera of the petaloid monocotyledons, *Allium* (Amaryllidaceae) comprises more than 920 species [1] and includes several economically important crops that are cultivated for consumption or medicinal uses, such as garlic (*A. sativum*), welsh onion (*A. fistulosum*), leek (*A. porrum*), Chinese chives (*A. tuberosum*), onion (*A. cepa*), Chinese jiaotou (*A. chinense*), and shallot (*A. ascalonicum*). Varied leaf shapes can be observed among *Allium* species, including flat, columnar, solid, and fistular morphologies. Morphological and cellular studies have found that fistular leaves develop from solid precursors [2]. Developmental investigation of the leaves of *A. fistulosum* found that the process of fistular leaf formation involved programmed cell death (PCD) [2]. PCD is a spontaneous, programmed, self-destructive cellular process that plays a key role in tissue differentiation, homeostasis, and organ morphogenesis, including that of leaves [3–5]. However, molecular evidence for the involvement of PCD in the formation of fistular leaf cavities is still absent in *Allium*, owing to the limited availability of genetic resources for *Allium* spp.

The paucity of genetic resources in *Allium* is, in part, due to the fact that *Allium* spp. have the largest genomes among eukaryotes [6, 7], with genome sizes ranging from 6860 to 30,870 Mbp per C [8]. The enormous size of these genomes has been a major obstacle for their characterization and for gene mining in the family as a whole. In the past 10 years, the next generation sequencing technologies have undergone rapid development, and more than 80 plant species have had their complete genome [9]. However, none of genome of *Allium* species has been characterized, because of their enormous size.

Because transcriptome analysis by next generation sequencing is rapid, inexpensive, and unconstrained by genomic complexity, it has been widely used as a primary tool for gene discovery and expression profiling in hundreds of plant species [10, 11]. Moreover, transcriptome analysis can also be used as an important tool for investigating the domesticated patterns of crops [12, 13], as well as for investigating the mechanisms of development for specific traits [14]. In *Allium*, the transcriptome of four species, i.e. garlic [15, 16], onion [17], Chinese chive [18], and welsh onion [19], have been sequenced and *de novo* assembled. Among these, onion and Welsh onion are fistular-leaved species, whereas the other two produce solid leaves. However, despite the large numbers of generated expressed sequence tags, the molecular mechanism for the formation of fistular leave cavities is still uncharacterized.

Therefore, we analyzed the transcriptomes of nine economically important *Allium* species, including eight vegetable species and one herbal species (*A. macrostemon*). Thereafter, genes that had undergone significant selection were identified in both fistular- and solid-leaf species, respectively, and the genes with evolutionary divergence between fistular- and solid-leaf species were screened. As a result, the current study presents molecular evidence regarding a potential evolutionary mechanism for the development of fistular leaves.

## Methods

### Plant material and RNA extraction

**Fig. 1:**
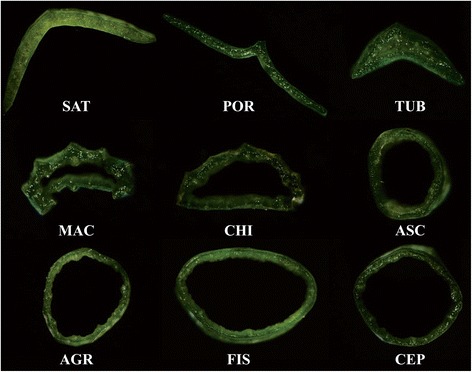
The transverse section picture of leaves of nine *Allium* species

The current study utilized local varieties of nine economically important *Allium* species. Among the eight vegetable species, three [*A. sativum* (garlic; SAT), *A. porrum* (leek; POR), and *A. tuberosum* (Chinese chives; TUB)] possess flat, solid leaves, whereas four species, *A. fistulosum* (welsh onion; FIS), *A. ascalonicum* (shallot; ASC), *A. cepa* (onion; CEP), and *A. cepa* var. *agrogarum* (AGR), have cylindrical, fistular leaves, and *A. chinense* (Chinese jiaotou; CHI) has triangular, fistular leaves (Fig. 1). In addition, the leaves of the herbal species *A. macrostemon* (MAC) are also fistular, but internal cavities are very small (Fig. 1). The varieties of SAT, CEP, and MAC were collected from Chaling (Hunan, China), whereas those of ASC, POR, TUB, and CHI were collected from Ningxiang (Changsha, China), and the varieties of FIS and AGR were collected from Yuanjiang (Hunan, China) and Fuyu (Jinlin, China), respectively. The transverse section of leaves of nine *Allium* species was observed by Nikon AZ100 microscope (Nikon, Toyota, Japan).

All the varieties were established in the experimental field of the Institute of Bast Fiber Crops, Chinese Academy of Agricultural Sciences, Changsha, China on Sept. 15, 2014, and on Mar. 10, 2015, leaf tissue was sampled from three individuals of each species, immediately frozen in liquid nitrogen, and stored at −80°C until used. The total RNA of each sample was extracted using an EZNA. Plant RNA Kit (OMEGA Bio-Tek, Norcross, GA, USA), according to the manufacturer’s protocol.

### cDNA library construction, sequencing, and assembly

Illumina sequencing for the nine species was performed at Novogene Bioinformatics Technology Co., Ltd (Beijing, China; www.novogene.com). First, the total RNA of each sample was used to construct a cDNA library with fragment lengths of ~250 bp. Thereafter, paired-end sequencing was performed using the Illumina HiSeq 2500 sequencing platform (Illumina, San Diego, CA, USA), and the clean sequencing reads of each species were deposited in the NCBI Sequence Read Archive (Additional file 1: Table S1). After trimming the adapter sequences and filtering low-quality reads, the reads of each species were used to assemble their transcriptomes *de novo* using Trinity [20]. Sequences not extended on either end were categorized as unigenes. The sequences of unigenes assembled are deposited in the NCBI Transcriptome Shotgun Assembly (TSA) Database (Additional file 1: Table S1).

### Gene annotation

To annotate the unigenes of each species, the corresponding sequences were searched against public databases, including the NCBI non-redundant protein sequences (NR) database, NCBI nucleotide sequences (NT) database, eukaryotic ortholog groups (KOG) database, Kyoto Encyclopedia of Genes and Genomes ortholog (KO) database, Swiss-Prot protein database, Gene Ontology (GO) database, and protein family (PFAM) database.

### Ortholog search and construction of phylogenetic tree

The OrthoMCL method, which is based on the Markov cluster algorithm [21], was used to identify for orthologous genes among the nine species. According to the orthologous gene sequences, the phylogenetic relationships of nine species were reconstructed using Phyml 3.0, which is based on a Bayes algorithm [22], and the tree was visualized using MEGA 4.0 [23].

### Ka/Ks analysis

Positive selection is a process that favors the retention of beneficial mutations, as well as the rapid diversification of the affected genes. If a gene has evolved under positive selection, the sequence is expected to contain more non-synonymous nucleotide substitutions (Ka) than synonymous nucleotide substitutions (Ks) and, accordingly, should also exhibit a high Ka/Ks ratio. In contrast, purifying selection is a process that removes deleterious alleles, and the sequences of genes subjected to purifying selection are relatively conserved. Accordingly, these sequences contain fewer, if any, non-synonymous substitutions, which results in a Ka that is much smaller than Ks, and a low Ka/Ks ratio. To identify genes subjected to significant selection, the Ka and Ks values were estimated using the Codeml program of Phylogenetic Analysis by Maximum Likelihood (PAML) based on the basic model [24], and genes with Ka/Ks ratios >1 were considered as under positive selection, whereas genes with Ka/Ks ratios <0.1 were considered as under purifying selection [25].

### Enrichment analysis

The enrichment of GO functional categories in the selected genes of each species were analyzed using GOseq, which is based on the Wallenius non-central hyper-geometric distribution [26], and enrichment analysis of the Kyoto Encyclopedia of Genes and Genomes (KEGG) pathways was performed using KOBAS [27]. Q values were used for determining the P-value threshold in multiple test and analysis [28], and pathways or GO categories with Q < 0.05 were considered significantly enriched.

## Results

### Transcriptome sequencing, assembly, and annotation

Illumina paired-end sequencing of leaf transcriptomes from nine *Allium* spp. yielded about 99.9–128.9 million clean sequencing reads with a mean length of ~125 bp for each species (Table 1). Except for AGR, in which only 83,186 unigenes were *de novo* assembled, all species yielded >117 thousand unigenes, with an average length of 529–641 bp. POR had the largest transcriptome, at 109.28 Mb, whereas the transcriptomes of AGR and CHI were relatively small, at 53.35 and 66.84 Mb, respectively, and the other six species possessed intermediate sized transcriptomes, ranging from 71.62 to 85.48 Mb (Table 1). In addition, except for the unigenes of POR and AGR, of which 25.62 and 41.04% were successfully annotated, respectively, ~30–35% of unigenes were typically annotated.

**Table 1:** Summary of transcriptome sequencing for nine *Allium* species

Species	Abbr.	Crop names	Leaf shape	Clean read number (Million)	Transcriptome size (Mb)	Unigene number	Mean length of unigene (bp)	Gene number annotated
*A. sativum*	SAT	Garlic	Flat solid leaf	103.6	77.95	132,225	590	46341 (35.04%)
*A. porrum*	POR	Leek	Flat solid leaf	128.9	109.28	189,713	576	48621 (25.62%)
*A. tuberosum*	TUB	Chinese chives	Flat solid leaf	109.4	83.78	148,715	563	44532 (29.94%)
*A. macrostemon*	MAC		Fistular leaf with small gap	99.9	85.48	161,681	529	52129 (32.24%)
*A. chinense*	CHI	Chinese jiaotou	Triangular fistular leaf	111.9	66.84	121,008	552	41908 (34.63%)
*A. ascalonicum*	ASC	Shallot	Columnar fistular leaf	100.8	71.62	117,659	609	40315 (34.26%)
*A. fistulosum*	FIS	Welsh onion	Columnar fistular leaf	121.0	76.56	128,372	596	42311 (32.95%)
*A. cepa* L. var. *cepa*	CEP	Onion	Columnar fistular leaf	102.9	74.03	117,189	632	39472 (33.68%)
*A. cepa* var. *agrogarum*	AGR		Columnar fistular leaf	102.5	53.35	83,186	641	34141 (41.04%)

### Phylogenetic analysis

**Fig. 2:**
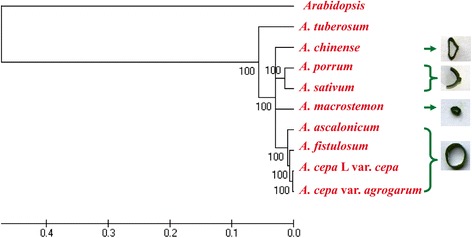
Phylogenetic tree of nine *Allium* species, constructed on the basis of their transcriptomes

According to our phylogenetic tree (Fig. 2), TUB was located at the root of the tree, and had far phylogenetic distance with the others, suggesting that TUB was more ancestral than the other species. This finding was consistent with the result of previous study [29]. The other eight species were split into four clades. Interestingly, the classification based on the transcriptomes was consistent with that based on leaf morphology, i.e., the species with flat, solid leaves (SAT and POR) were grouped together, the species with cylindrical, fistular leaves (ASC, FIS, CEP, and AGR) were placed in a monophyletic clade, and the species with small-gapped and triangular fistular leaves (MAC and CHI) were placed in a single branch, as well (Fig. 2).

### Identification of genes subjected to significant selection

**Fig. 3:**
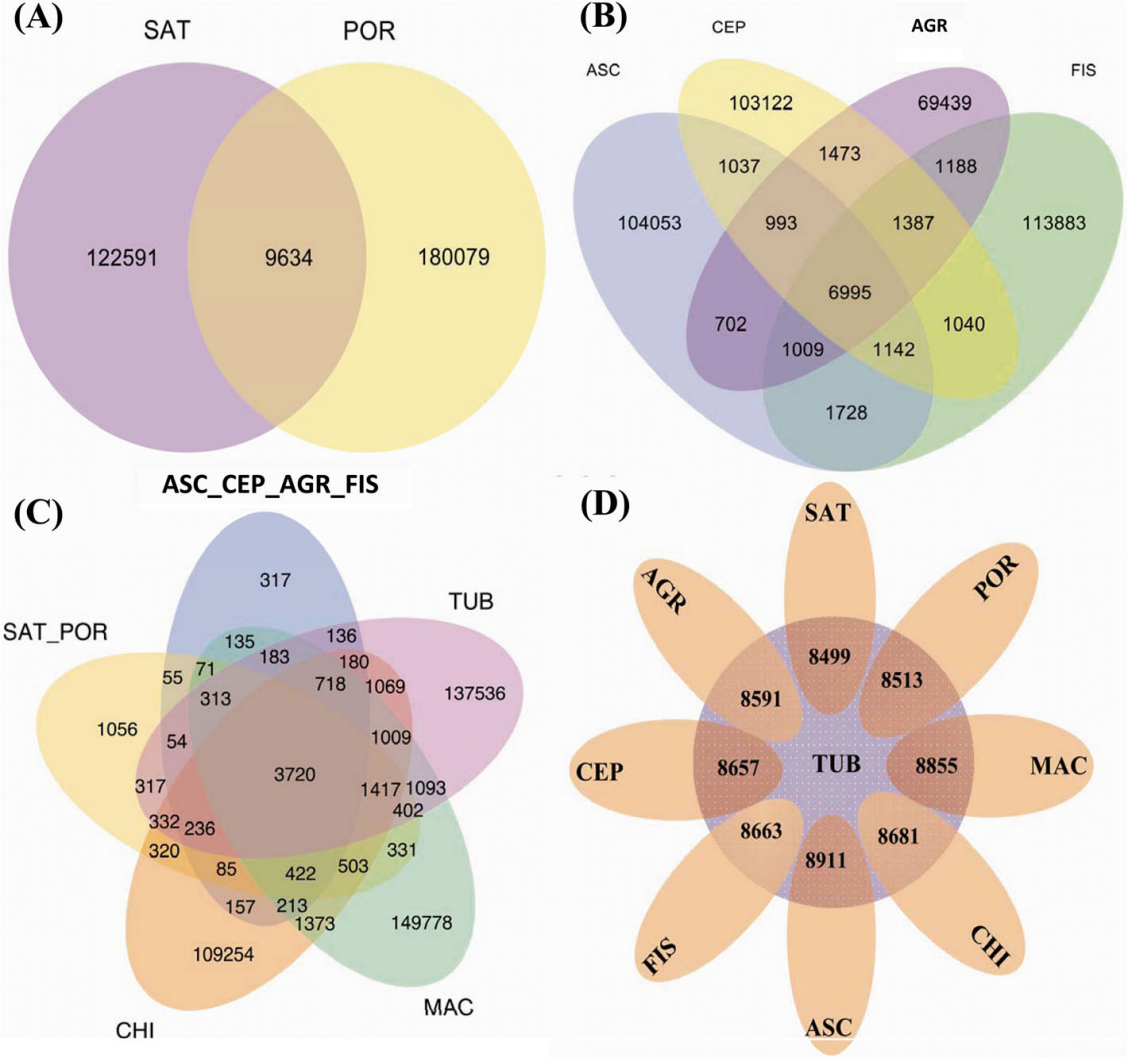
Venn diagram of the orthologous genes in nine *Allium* species. **a** The orthologs of two solid-leaved species: SAT and POR; **b** the orthologs of four columnar fistular-leaved species; **c** the orthologs of all nine species, in which SAT_POR and ASC_CEP_AGR_FIS represent the orthologs of SAT and POR and the orthologs of ASC, CEP, AGR, and FIS, respectively; **d** the orthologs of TUB and the other eight species

A total of 29,990 orthologous groups were identified, with 9634 from the two flat, solid-leaved species (SAT and POR; Fig. 3a), 6995 from the four cylindrical, fistular-leaved species (ASC, FIS, CEP, and AGR; Fig. 3b), 3720 among all nine species (Fig. 3c).

**Fig. 4:**
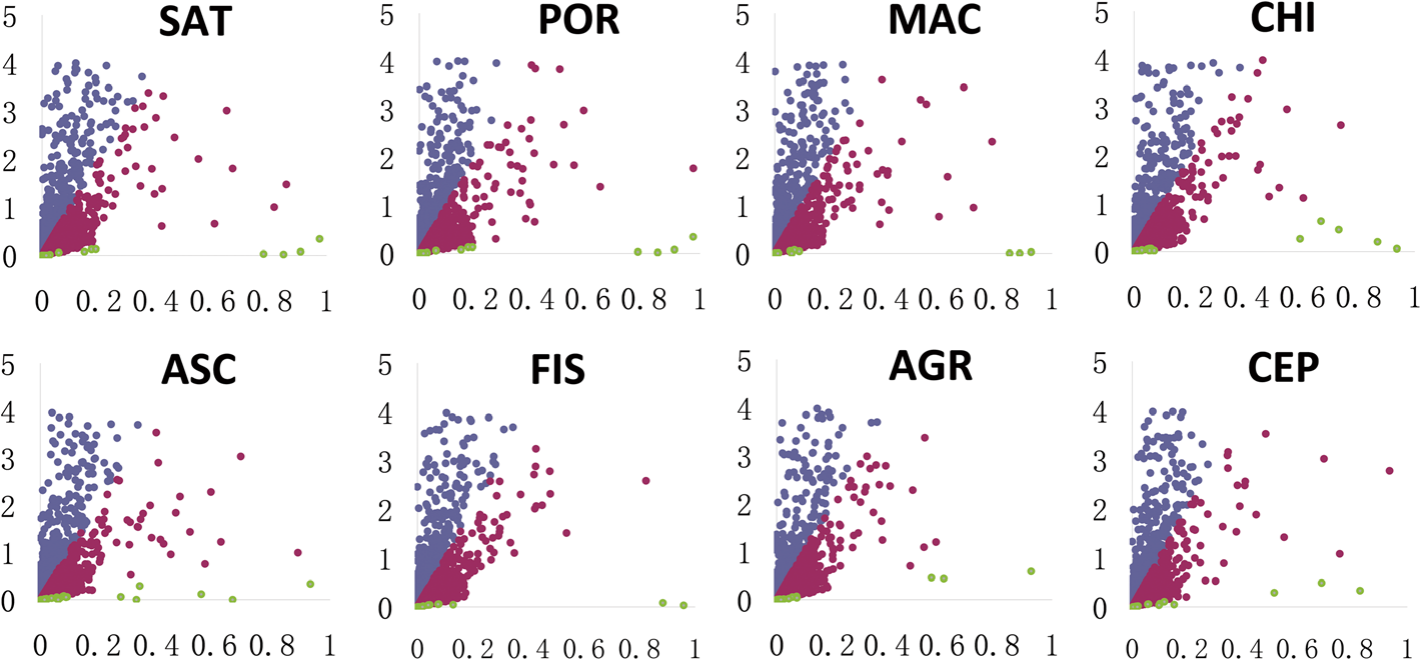
Scatter diagrams of Ka and Ks values. The Ka and Ks values of the orthologs of TUB and the other eight species were estimated. The *x* and *y* axes represent the Ka and Ks values, respectively. Orthologous genes with Ka > 1 or Ks > 4 are not shown

**Fig. 5:**
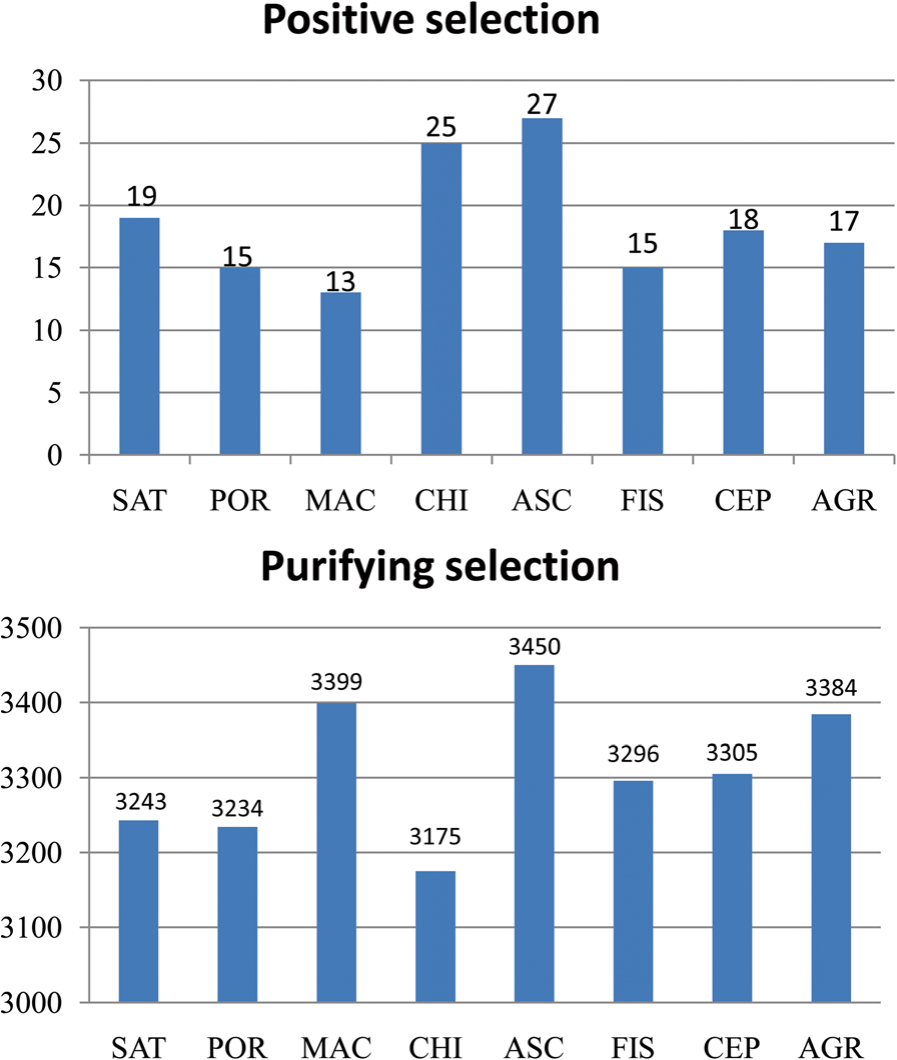
The gene number subjected to positive and purifying selection in each species

Because the TUB transcriptome was ancestral to the transcriptomes of the other eight species, it was designated as the ancestral species in the present study. The orthologous genes between TUB and the other eight species were searched (Fig. 3d), and their Ka and Ks values were estimated and used to determine the selective pattern of them (Fig. 4). A total of 149 genes were found to undergo positive selection with 13 to 27 identified in each species (Fig. 5), suggesting that these 149 genes exhibited a rapid diversification in evolutionary history. Among these 149 positively selected genes, 39 genes (from 18 orthologous groups) were mitochondrial (Additional file 2: Table S2), 29 genes (from 13 orthologous groups) were chloroplast (Additional file 3: Table S3), and 81 genes (from 70 orthologous groups) were nuclear (Additional file 4: Table S4). In addition, >3000 genes were found to undergo purifying selection in each species (Fig. 5), which suggested that the sequences of these genes were relatively conserved, in evolutionary history.

### Enrichment analysis for selected genes

None of the GO categories were enriched by positively selected genes in any species, although 51–72 terms were significantly enriched by genes subjected to purifying selection in each species (Q < 0.05; Additional file 5: Table S5). Among the enriched terms, 23 were enriched by genes of purifying selection in all eight species, suggesting that the functional terms are essential for growth in the species. In addition, one KEGG pathway, oxidative phosphorylation, exhibited significant enrichment by positively selected genes in all species, except FIS (Q < 0.05); however, no pathways were markedly enriched by genes of purifying selection (Q < 0.05).

### Identification of selected genes potentially related to PCD

Among 149 identified positively selected genes, 25 from 11 orthologous groups were annotated as proteins involved in plant PCD (Table 2), including 15 genes related to the metabolism of cytochrome c, which is an important factor for inducing PCD [30], and two genes encoding PCD-related kinase proteins. In addition, there were 4970 orthologous groups in which the orthologous gene of at least two species had undergone purifying selection. Of the 4,970 orthologous groups, there were 116 groups in which only the orthologous genes from the flat, solid-leaved species(SAT and POR) were subjected to purifying selection (Additional file 6: Table S6), suggesting that the genes are possibly related to leaf morphology. Among the 116 orthologous groups, five were PCD-related (Table 3), especially OG01095 that encodes a metacaspase-like protein, which is a key regulator of plant PCD [31].

**Table 2:** Positively selected genes potentially related to programmed cell death

Orthologous group	Positively selected genes	Gene source	Function annotated
OG06438	MAC|c99454_g1, CHI|c73660_g1, ASC|c61655_g1, FIS|c70920_g1, CEP|c54051_g1, AGR|c38049_g1	Mitochondrial genome	Cytochrome c oxidase subunit 3
OG08411	MAC|c48312_g1, ASC|c92926_g1	Mitochondrial genome	cytochrome c biogenesis Fn
OG12823	ASC|c67719_g2, AGR|c41049_g1	Mitochondrial genome	cytochrome c oxidase subunit 2
OG13835	SAT|c125661_g1, POR|c94489_g1, MAC|c79087_g1	Mitochondrial genome	cytochrome c maturation protein CcmB
OG20657	CEP|c5005_g1, AGR|c29721_g1	Mitochondrial genome	cytochrome c biosynthesis ccmC-like protein
OG04672	SAT|c121207_g1, MAC|c150852_g1, ASC|c70366_g1, FIS|c65166_g1	Chloroplastic Genome	photosystem II protein D1
OG27563	ASC|c29090_g1	Chloroplastic Genome	photosystem II protein
OG15984	FIS|c46048_g1, CEP|c60376_g1	Nuclear Genome	Serine/threonine-protein kinase ATM-like protein
OG22529	CHI|c103126_g1	Nuclear Genome	serine/threonine-protein kinase ATR-like protein
OG28738	ASC|c79214_g1	Nuclear Genome	KDEL-tailed cysteine endopeptidase
OG09355	FIS|c74337_g2	Nuclear Genome	LOL2-like protein

**Table 3:** Programmed cell death-related orthologous groups in which only the orthologous genes from the flat, solid-leaved species *A. sativum* and *A. porrum* were subjected to purifying selection

Orthologous group	Genes of purifying selection	Gene source	Function annotated
OG02476	POR|c97456_g1, SAT|c68586_g1	Nuclear Genome	mitogen-activated protein kinase 9-like
OG01095	SAT|c13216_g1, POR|c105213_g1	Nuclear Genome	metacaspase-4-like
OG03448	SAT|c76384_g1, POR|c107735_g1	Nuclear Genome	MACPF domain-containing protein CAD1
OG10721	SAT|c83301_g1, POR|c11731_g1	Chloroplastic Genome	photosystem II protein, chloroplast
OG23573	SAT|c138076_g1, POR|c7088_g1	Chloroplastic Genome	photosystem II P680 reaction center D1 protein, chloroplast

## Discussion

Although *Allium* comprises a large number of species and even includes a few species of significant economic importance, the transcriptomes of only four species (i.e. garlic, onion, welsh onion, and Chinese chive) have been analyzed [15–19]. Therefore, in the present study, the transcriptomes of nine *Allium* species were sequenced and assembled *de novo*, and large numbers of genes were identified in each species. These results will help us investigate the major genes responsible for important agronomic traits in *Allium* species, and will also further our understanding of the mechanisms underlying the formation and development of such traits, including leaf morphology.

Leaves are the primary site of plant photosynthesis and respiration. Diverse leaf forms can be observed in angiosperms, and they can be categorized into two major categories: bifacial or unifacial. Development studies have proposed that the diversity of leaf morphology is caused by differential and asymmetrical growth along the adaxial–abaxial, central–marginal, and proximal–distal polarities [32–34]. *Allium* spp. exhibit a wide variety of leaf shapes, including cylindrical and hollow, flat and solid, and hollow and triangular types, the differences of which can be observed in transverse sections [35]. As in other angiosperms, there is a great possibility that the diversity of leaf appearance in *Allium* is caused by these three polarities. For example, only the abaxial identity existed in leaf blades results in a unifacial fistular leaf in Welsh onion [33, 34] However, the formation of fistular leaf cavities is still poorly understood. Although that a cellular investigation of the leaves of *A. fistulosum* found that the process of fistular leaf formation involved PCD [2], molecular evidences for the involvement of PCD in the formation of fistular leaf cavities is still unknown in *Allium*.

Previous studies have shown that mitochondria play a central role in controlling PCD in plants [30], by releasing molecules, including cytochrome c that drive the destruction of the cell [36]. In animals, cytochrome c triggers PCD by forming a complex that induces the formation of apoptosomes, and those apoptosomes then activate downstream effector caspases, which are key regulators of PCD [30]. Although no caspase homologs have been found in plants, caspase-like proteins have been described in cells undergoing PCD, such as the cysteine endopeptidases, which can be subdivided into two groups: vacuolar processing enzymes and metacaspases [37, 38]. In the present study, we identified 15 positively selected genes from five orthologous groups that were potentially related to the metabolism of cytochrome c and were found to undergo positive selection. Among these 15 genes, 12 were subjected positive selection in fistular-leaved species, except 3 genes from OG13835 that underwent positive selection in the species with solid or small-gapped fistular leaves. In addition, a KDEL-tailed cysteine endopeptidase-encoding gene from the fistular-leaved ASC exhibited rapid evolution, and a metacaspase-4-like protein-encoding gene was found to undergo positive selection in solid-leaved species, but evolved neutrally in fistular-leaved species.

Like the mitochondria, the chloroplasts are also important in triggering PCD, by mediating reactive oxygen species signaling. In plants, photosynthesis is performed in photosystem II of chloroplasts. When photosynthesis is less efficient, or chloroplasts receive excessive levels of light, chloroplasts produce more reactive oxygen species, and then the reactive oxygen species drives cell death finally [30, 39, 40]. In the present study, we identified five chloroplast genes that are potentially involved in photosynthesis in photosystem II and exhibited rapid diversification. Among them, four of the genes were from fistular-leaved species, and one was from a flat, solid-leaved species (i.e., SAT). In addition, two photosystem II protein-encoding genes were also found to have undergone purfying selection in solid-leaved species, whereas they were evolving neutrally in fistular-leaved species.

After the “death signal” is generated by mitochondria and/or chloroplasts, it is transduced by kinase proteins. Previous studies have shown that PCD signaling involves the activation of mitogen-activated protein kinases that induce hypersensitive responses and cell apoptosis [41]. Recently, the phosphatidyl-3 kinases ATM and ATR were reported as the main sensors of DNA damage-induced PCD [42, 43]. In the present study, the ATM and ATR kinase-encoding genes in the fistular-leaved species FIS, CEP, and CHI exhibited evidence of rapid diversification, but not in solid-leaved species, and mitogen-activated protein kinase-encoding gene underwent positive selection in solid-leaved species, whereas it was evolving neutrally in fistular-leaved species.

In addition, two genes, *LSD-one-like protein 2* (*LOL2*) and *constitutively activated cell death 1* (*CAD1*), have also been reported to play important roles in regulating PCD in *Arabidopsis* [44, 45]. In the present study, the orthologs of these two genes were also found to exhibit different selective patterns among the fistular- and solid-leaved species. These potential PCD-related genes with evolutionary divergence might have roles in regulating the formation of cavity of fistular leaf in *Allium* species.

## Conclusion

In this study, we compared the leaf transcriptomes of six fistular- and three solid-leaf species of *Allium*. The selective patterns of orthologous genes in nine species were analyzed, and the result showed that many mitochondrial, chloroplast and nuclear genes that were potentially involved in plant PCD and were either subject to positive selection in fistular-leaved species but not in solid-leaved species, or evolved neutrally in fistular-leaved species but purifying selection in solid-leaved species. These potential PCD-related genes might play roles in the formation of the inner cavities of fistular leaves in *Allium*, and the differing selection patterns in fistular- and solid-leaved species may be responsible for the evolution of fistular leaves.

## Additional files

Additional file 1: Table S1. Total length of clean sequencing reads from each species and their accession numbers in database. (DOCX 16 kb)
Additional file 2: Table S2. Positively selected mitochondrial genes in evolution. (DOCX 13 kb)
Additional file 3: Table S3. Positively selected chloroplast genes in evolution. (DOCX 13 kb)
Additional file 4: Table S4. Positively selected nuclear genes in evolution. (XLSX 14 kb)
Additional file 5: Table S5. Table S5 Gene ontology (GO) terms enriched by genes of purifying selection in each species. (XLSX 16 kb)
Additional file 6: Table S6. Orthologous groups in which only the orthologous genes from the flat, solid-leaved species *A. sativum* and *A. porrum* were subjected to purifying selection*. (XLSX 17 kb)*

## Abbreviations

AGR: *A. cepa* var. *agrogarum*
ASC: *A. ascalonicum*
CEP: *A. cepa*
CHI: *A. chinense*
FIS: *A. fistulosum*
GO: Gene Ontology database
Ka: Non-synonymous nucleotide substitutions
KO: KEGG ortholog database
KOG: Eukaryotic ortholog groups database
Ks: Synonymous nucleotide substitutions
MAC: *A. macrostemon*
NR: NCBI non-redundant protein sequencesdatabase
NT: NCBI nucleotide sequences database
PCD: Programmed cell death
PFAM: Protein family database
POR: *A. porrum*
SAT: *A. sativum*
TUB: *A. tuberosum*

## References

[1] Herden T, Hanelt P, Friesen N (2016). Phylogeny of Allium L. subgenus Anguinum (G. Don. ex W.D.J. Koch) N. Friesen (Amaryllidaceae). Mol Phylogenet Evol.

[2] Ni X, Su H, Zhou Y, Wang F, Liu W (2015). Leaf-shape remodeling: programmed cell death in fistular leaves of *Allium fistulosum*. Physiol Plant.

[3] Meier P, Finch A, Evan G (2000). Apoptosis in development. Nature.

[4] Gunawardena A, Greenwood J, Dengler N (2004). Programmed cell death remodels lace plant leaf shape during development. Plant Cell.

[5] Gunawardena A, Sault K, Donnelly P, Greenwood J, Dengler N (2005). Programmed cell death and leaf morphogenesis in *Monstera obliqua* (Araceae). Planta.

[6] Labani R, Elkington T (1987). Nuclear DNA variation in the genus *Allium* L. (Liliaceae). Heredity.

[7] Baranyi M, Greilhuber J (1999). Genome size in *Allium*: in quest of reproducible data. Ann Bot.

[8] Ricroch A, Yockteng R, Brown SC, Nadot S (2005). Evolution of genome size across some cultivated *Allium* species. Genome.

[9] Kitts P, Church D, Thibaud-Nissen F, Choi J, Hem V, Sapojnikov V, Smith R, Tatusova T, Xiang C, Zherikov A (2016). Assembly: a resource for assembled genomes at NCBI. Nucleic Acids Res.

[10] Liu T, Zhu S, Tang Q, Chen P, Yu Y, Tang S (2013). *De novo* assembly and characterization of transcriptome using Illumina paired-end sequencing and identification of *CesA* gene in ramie (*Boehmeria nivea* L. Gaud). BMC Genomics.

[11] Mutz K, Heilkenbrinker A, Lonne M, Walter J, Stahl F (2013). Transcriptome analysis using next-generation sequencing. Curr Opin Biotechnol.

[12] Liu T, Tang S, Zhu S, Tang Q, Zheng X (2014). Transcriptome comparison reveals the patterns of selection in domesticated and wild ramie (*Boehmeria nivea* L. Gaud). Plant Mol Biol.

[13] Koenig D, Jiménez-Gómez J, Kimura S, Fulopa D, Chitwooda D, Headlanda L, Kumara R, Covingtona M, Devisettya U, Tata A (2013). Comparative transcriptomics reveals patterns of selection in domesticated and wild tomato. Proc Natl Acad Sci U S A.

[14] Lou Q, Liu Y, Qi Y, Jiao S, Tian F, Jiang L, Wang Y (2014). Transcriptome sequencing and metabolite analysis reveals the role of delphinidin metabolism in flower colour in grape hyacinth. J Exp Bot.

[15] Kamenetsky R, Faigenboim A, Mayer E, Michael T, Gershberg C, Kimhi S, Esquira I, Shalom S, Eshel D, Rabinowitch H (2015). Integrated transcriptome catalogue and organspecific profiling of gene expression in fertile garlic (*Allium sativum* L.). BMC Genomics.

[16] Liu T, Zeng L, Zhu S, Chen X, Tang Q, Mei S, Tang S (2015). Large-scale development of expressed sequence tag-derived simple sequence repeat markers by deep transcriptome sequencing in garlic (*Allium sativum* L.). Mol Breed.

[17] Rajkumar H, Ramagoni R, Anchoju V, Vankudavath R, Syed A (2015). *De novo* transcriptome analysis of *Allium cepa* L. (Onion) bulb to identify allergens and epitopes. PLoS One.

[18] Zhou S, Chen L, Liu S, Wang X, Sun X (2015). *De novo* assembly and annotation of the Chinese chive (*Allium tuberosum* Rottler ex Spr.) transcriptome using the Illumina platform. PLoS One.

[19] Sun X, Yu X, Zhou S, Liu S (2016). *De novo* assembly and characterization of the Welsh onion (*Allium fistulosum* L.) transcriptome using Illumina technology. Mol Genet Genomics.

[20] Grabherr M, Haas B, Yassour M, Levin J, Thompson D, Amit I, Adiconis X, Fan L, Raychowdhury R, Zeng Q (2011). Full length transcriptome assembly from RNA-Seq data without a reference genome. Nat Biotechnol.

[21] Li L, Stoeckert C, Roos D (2003). OrthoMCL: Identification of ortholog groups for eukaryotic genomes. Genome Res.

[22] Guindon S, Dufayard J, Lefort V, Anisimova M, Hordijk W, Gascuel O (2010). New algorithms and methods to estimate maximum-likelihood phylogenies: assessing the performance of PhyML 3.0.. Syst Biol.

[23] Tamura K, Dudley J, Nei M, Kumar S (2007). MEGA4: molecular evolutionary genetics analysis (MEGA) software version 4.0.. Mol Biol Evol.

[24] Yang Z (2007). PAML 4: Phylogenetic analysis by maximum likelihood. Mol Biol Evol.

[25] Shah C, VanGompel MJW, Naeem V, Chen Y, Lee T, Angeloni N, Wang Y, Xu E (2010). Widespread presence of human *BOULE* homologs among animals and conservation of their ancient reproductive function. PLoS Genet.

[26] Young M, Wakefield M, Smyth G, Oshlack A (2010). Gene ontology analysis for RNA-seq: accounting for selection bias. Genome Biol.

[27] Xie C, Mao X, Huang J, Ding Y, Wu J, Dong S, Kong L, Gao G, Li C, Wei L (2011). KOBAS 2.0: a web server for annotation and identification of enriched pathways and diseases. Nucleic Acids Res.

[28] Benjamini Y, Hochberg Y (1995). Controlling the false discovery rate: a practical and powerful approach to multiple testing. J Roy Stat Soc B.

[29] Friesen N, Fritsch R, Blattner F (2006). Phylogeny and new intrageneric classification of *Allium* (Alliaceae) based on nuclear ribosomal DNA ITS sequences. Aliso.

[30] Reape T, Brogan N, McCabe P, Gunawardena A, McCabe P (2015). Mitochondrion and chloroplast regulation of plant programmed cell death. Plant Programmed Cell Death.

[31] Lam E, Zhang Y (2012). Regulating the reapers: activating metacaspases for programmed cell death. Trends Plant Sci.

[32] Yamaguchi T, Tsukaya H (2010). Evolutionary and developmenta studies of unifacial leaves in monocots: Juncus as a model system. J Plant Res.

[33] Yamaguchi T, Nukazuka A, Tsukaya H (2012). Leaf adaxial–abaxial polarity specification and lamina outgrowth: evolution and development. Plant Cell Physiol.

[34] Yamaguchi T, Yano S, Tsukaya H (2010). Genetic framework for flattened leaf blade formation in unifacial leaves of Juncus prismatocarpus. Plant Cell.

[35] Lee SH (1952). A comparative anatomical study on the leaves of some bulb crops in China. J Integr Plant Biol.

[36] Diamond M, McCabe P, Kempken F (2011). Mitochondrial regulation of plant programmed cell death. Plant mitochondria, vol 1, Advances in plant biology.

[37] Helm M, Schmid M, Hierl G, Terneus K, Tan L, Lottspeich F, Kieliszewski M, Gietl C (2008). KDEL-tailed cysteine endopeptidases involved in programmed cell death, intercalation of new cells, and dismantling of extension scaffolds. Am J Bot.

[38] Lord C, Dauphinee A, Watts R, Gunawardena. A unveiling interactions among mitochondria, caspase-Like proteases, and the actin cytoskeleton during plant programmed cell death (PCD). PLoS ONE. 2013;8:e57110. 10.1371/journal.pone.0057110 PMC3590192 23483897

[39] Kim C, Meskauskiene R, Zhang S, Lee K, Ashok M, Blajecka K, Herrfurth C, Feussner I, Apela K (2012). Chloroplasts of *Arabidopsis* are the source and a primary target of a plant-specific programmed cell death signaling pathway. Plant Cell.

[40] Aken O, Breusegem F (2015). Licensed to kill: mitochondria, chloroplasts, and cell death. Trends Plant Sci.

[41] Kuriyama H, Fukuda H (2002). Developmental programmed cell death in plants. Curr Opin Plant Biol.

[42] Amiard S, Depeiges A, Allain E, White C, Gallego M (2011). *Arabidopsis* ATM and ATR kinases prevent propagation of genome damage caused by telomere dysfunction. Plant Cell.

[43] Smetana O, Siroky J, Houlne G, Opatrny Z, Chaboute M (2012). Non-apoptotic programmed cell death with paraptotic-like features in bleomycin-treated plant cells is suppressed by inhibition of ATM/ATR pathways or *Nt*E2F overexpression. J Exp Bot.

[44] Morita-Yamamuro C, Tsutsui T, Sato M, Yoshioka H, Tamaoki M, Ogawa D, Matsuura H, Yoshihara T, Ikeda A, Uyeda I (2005). The *Arabidopsis* gene *CAD1* controls programmed cell death in the plant immune system and encodes a protein containing a MACPF domain. Plant Cell Physiol.

[45] Coll N, Epple P, Dangl J (2011). Programmed cell death in the plant immune system. Cell Death Differ.

