# Supplementary material for: Comparative transcriptomics provide insight into the morphogenesis and evolution of fistular leaves in Allium
Source: BMC Genomics. 2017 Jan 10;18:60. doi: 10.1186/s12864-016-3474-8 (PMC5223570; doi:10.1186/s12864-016-3474-8)
Supplement: Additional file 1: Table S1. — Total length of clean sequencing reads from each species and their accession numbers in database. (DOCX 16 kb) [file 12864_2016_3474_MOESM1_ESM.docx]

Table S1 Total length of clean sequencing reads from each species and their accession numbers in database

| Species | Crop name | Size of Clean sequencing reads | Accession number in SRA database | Accession number in TSA database |
| --- | --- | --- | --- | --- |
| A. sativum | garlic | 12.94 | SRX1560526 | - GFAP00000000 |
| A. porrum | leek | 16.12 | SRX1560538 | - GFAR00000000 |
| A. tuberosum | chinese chives | 13.68 | SRX1560563 | - GFAN00000000 |
| A. macrostemon |  | 12.48 | SRX1560658 | - GFAO00000000 |
| A. chinense | chinese jiaotou | 13.98 | SRX1560673 | - GFAL00000000 |
| A. ascalonicum | shallot | 12.6 | SRX1560692 | - GFAJ00000000 |
| A. cepa L var. cepa | onion | 12.86 | SRX1560712 | GEOY00000000 |
| A. cepa var.agrogarum |  | 12.8 | SRX1560726 | - GFAK00000000 |
| A. fistulosum | welsh onion | 15.12 | SRX1560727 | - GFAM00000000 |
