# Supplementary material for: Comparative transcriptomics provide insight into the morphogenesis and evolution of fistular leaves in Allium
Source: BMC Genomics. 2017 Jan 10;18:60. doi: 10.1186/s12864-016-3474-8 (PMC5223570; doi:10.1186/s12864-016-3474-8)
Supplement: Additional file 2: Table S2. — Positively selected mitochondrial genes in evolution. (DOCX 13 kb) [file 12864_2016_3474_MOESM2_ESM.docx]

Table S2 Positively selected mitochondrial genes in evolution

| Orthologous group | Positively selected genes | Function annotated |
| --- | --- | --- |
| OG02566 | CEP\|c65615_g3, AGR\|c34913_g1 | ATP synthase subunit 9 |
| OG06438 | MAC\|c99454_g1, CHI\|c73660_g1, ASC\|c61655_g1, FIS\|c70920_g1, CEP\|c54051_g1, AGR\|c38049_g1 | Cytochrome c oxidase subunit 3 |
| OG08410 | CHI\|c82632_g1, ASC\|c69182_g5, AGR\|c41308_g1 | ATP synthase subunit 6 |
| OG08411 | MAC\|c48312_g1, ASC\|c92926_g1 | cytochrome c biogenesis Fn |
| OG09617 | SAT\|c82711_g3, POR\|c107112_g1, MAC\|c109945_g1, ASC\|c68822_g1, FIS\|c61401_g1, CEP\|c48006_g1, AGR\|c29615_g1 | NADH dehydrogenase subunit 9 |
| OG12823 | ASC\|c67719_g2, AGR\|c41049_g1 | cytochrome c oxidase subunit 2 |
| OG13835 | SAT\|c125661_g1, POR\|c94489_g1, MAC\|c79087_g1 | cytochrome c maturation protein CcmB |
| OG17660 | ASC\|c68646_g3, CEP\|c41981_g1 | uncharacterized protein |
| OG18472 | CHI\|c42294_g1, AGR\|c76284_g1 | hypothetical protein |
| OG20657 | CEP\|c5005_g1, AGR\|c29721_g1 | cytochrome c biosynthesis ccmC-like protein |
| OG15219 | ASC\|c115922_g1 | hypothetical protein |
| OG28409 | ASC\|c69182_g2 | NADH-ubiquinone oxidoreductase chain |
| OG22167 | CEP\|c63693_g2 | NADH dehydrogenase subunit 1 |
| OG10724 | FIS\|c65055_g1 | ATP synthase subunit b |
| OG04674 | CHI\|c80457_g2 | NADH dehydrogenase subunit 7 |
| OG14631 | CHI\|c63409_g1 | NADH dehydrogenase subunit 3, partial |
| OG15140 | MAC\|c134603_g1 | hypothetical protein |
| OG02425 | SAT\|c80565_g2 | NADH dehydrogenase subunit 4 |
