# Supplementary material for: Comparative transcriptomics provide insight into the morphogenesis and evolution of fistular leaves in Allium
Source: BMC Genomics. 2017 Jan 10;18:60. doi: 10.1186/s12864-016-3474-8 (PMC5223570; doi:10.1186/s12864-016-3474-8)
Supplement: Additional file 3: Table S3. — Positively selected chloroplast genes in evolution. (DOCX 13 kb) [file 12864_2016_3474_MOESM3_ESM.docx]

Table S3 Positively selected chloroplast genes in evolution

| Orthologous group | Positively selected genes | Function annotated |
| --- | --- | --- |
| OG04543 | FIS\|c107223_g1, CEP\|c40560_g1 | ribosomal protein S14 |
| OG04672 | SAT\|c121207_g1, MAC\|c150852_g1, ASC\|c70366_g1, FIS\|c65166_g1 | photosystem II protein D1 |
| OG05548 | SAT\|c86777_g1, POR\|c73015_g1, MAC\|c176429_g1, ASC\|c4764_g1, FIS\|c36784_g1, AGR\|c11496_g1 | ribosomal protein S18 |
| OG13914 | POR\|c167294_g1, CHI\|c104656_g1, ASC\|c1238_g1, CEP\|c67203_g1, AGR\|c22087_g1 | clp protease proteolytic subunit |
| OG14158 | SAT\|c76333_g1, CEP\|c110945_g1 | hypothetical protein |
| OG16943 | POR\|c33729_g1, ASC\|c104035_g1, AGR\|c5426_g1 | ORF411 |
| OG27563 | ASC\|c29090_g1 | Ycf5 |
| OG29550 | CEP\|c22683_g2 | orf137 |
| OG33014 | FIS\|c71175_g4 | ycf2, partial |
| OG15122 | CHI\|c75974_g1 | Protein Ycf2 |
| OG35149 | MAC\|c9514_g1 | hypothetical protein |
| OG15123 | POR\|c162104_g1 | NADH-plastoquinone oxidoreductase subunit I |
| OG00907 | SAT\|c104465_g1 | RNA polymerase beta' subunit |
